# Senescent cells: A therapeutic target for osteoporosis

**DOI:** 10.1111/cpr.13323

**Published:** 2022-08-19

**Authors:** Tiantian Wang, Shishu Huang, Chengqi He

**Affiliations:** ^1^ Department of Rehabilitation Medicine, Key Laboratory of Rehabilitation Medicine, West China Hospital Sichuan University Chengdu Sichuan China; ^2^ Institute of Rehabilitation Medicine, West China Hospital Sichuan University Chengdu Sichuan China; ^3^ Department of Orthopedic Surgery and Orthopedic Research Institute, West China Hospital and West China School of Medicine Sichuan University Chengdu Sichuan China

## Abstract

**Background:**

Osteoporosis (OP) is a prevalent disorder characterized by the loss of bone mass and the deterioration of bone microarchitecture. OP is attributed to various factors, including menopause (primary), ageing (primary) and the adverse effects of medications (secondary). Recently, cellular senescence has been shown to have a crucial role in the maintenance of cellular homeostasis and organ function. The purpose of this review is to summarize recent findings regarding the roles of bone cellular senescence and senescence‐associated secretory phenotype (SASP) in OP.

**Methods:**

A comprehensive search of the PubMed database from inception to July 2022 was performed regarding the molecular mechanism of bone cell senescence in OP progression.

**Results:**

We describe the pathophysiology of senescent bone cells and SASP, and how each contributes to OP. We also provide new options for treating OP by targeting cellular senescence pathways.

**Conclusion:**

Cellular senescence plays an important role in bone homeostasis, with variations based on the different types of OP. These variations are associated with pathogenic factors, bone turnover rate and systemic metabolism. Understanding the molecular relationship between bone cells and senescence provides for the possible targeting of senescence as a means by which to treat OP.

## Graphical abstract

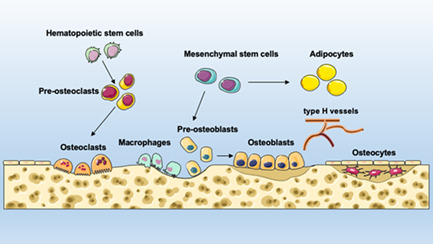

## 1 INTRODUCTION

Osteoporosis (OP) is the classic cause of low bone mass and is characterized by the deterioration of bone tissue, which increases bone fragility and leads to other serious conditions resultant from low bone mineral density (BMD).^1^, ^2^ In Europe, nearly 22 million women and 5.5 million men are estimated to suffer from OP.^3^ In the United States, 10 million individuals are estimated to have OP, with the expectation that this number will continue to increase.^4^ Ageing is closely related to OP in individuals over 50 years of age.^5^ Oestrogen deficiency is a common cause of primary OP, especially among postmenopausal women. Secondary OP is induced by certain medications and medical conditions.^5^, ^6^, ^7^ For example, glucocorticoid‐induced OP (GIOP) caused by long‐term usage of glucocorticoids (GCs) is the most common form of secondary OP.^7^, ^8^ Disease pathology is complex, heterogeneous and ill identified. Moreover, the underlying causative disease mechanism is not fully understood, and no disease‐modifying treatments are currently available.

Cellular senescence is a cell state implicated in various physiological processes and has been associated with a wide range of age‐related diseases,^9^ with senescent cells and heterogeneous cellular states associated with senescence‐associated secretory phenotype (SASP) of particular interest. Targeting senescent bone cells and SASP has been shown to alleviate OP.^10^, ^11^, ^12^, ^13^ Although promising, the mechanistic relationships among cellular senescence, SASP and OP pathology are unclear.^14^, ^15^, ^16^, ^17^, ^18^

The purpose of this review is to summarize recent findings regarding the roles of bone cellular senescence and SASP in OP. We discuss results demonstrating the effect of cellular senescence alteration on primary and secondary OP, which may provide new translational medicine options for treating OP.

## 2 BIOLOGICAL ROLES FOR CELLULAR SENESCENCE

The term ‘senescence’ was first described by Hayflick and Moorhead over 50 years ago, when normal human fibroblasts lost replicative potential, only remaining alive and metabolically active for approximately 50 cellular divisions.^19^, ^20^ To date, replicative senescence has been observed after multiple cell divisions of normal cells. The shortening of telomeres as a consequence of multiple cell divisions of non‐transformed cells contributes to this type of senescence.^21^, ^22^ After several cellular divisions, telomeres become critically short and no longer protect structural DNA, which initiates DNA damage. Response to DNA damage arrests the cell cycle, due to posttranslational modification of several cell cycle proteins related to cellular senescence.^21^, ^22^ In addition to replicative senescence, another type of senescence termed, stress‐induced premature senescence (SIPS), is caused by other stimuli, such as DNA damage, oxidative stress, toxins, hyperglycaemia, inflammation and ultraviolet radiation.^23^, ^24^, ^25^ Persistent stress induces cells to lose their ability to repair DNA, which causes permanent cell cycle arrest.^26^, ^27^ Two major pathways, the p53/p21 and p16INK4a/retinoblastoma (Rb) pathways, contribute to senescence.^28^ For example, blockade of p53 function in senescent human fibroblasts induces a reversion to a ‘young’ morphology.^29^ Inactivation of p21, a cell cycle inhibitor targeted to p53, facilitates normal diploid human fibroblasts to bypass senescence despite the expression of p16.^30^ Further, accelerated clearance of p16INK4a‐positive senescent cells in various mouse tissues has been shown to reduce age‐related pathologies including preservation of muscle function as well as decreased eye senescence. These results suggest that activation of the p16 signalling pathway directly contributes to senescence and tissue degeneration.^31^

Transient induction of cellular senescence has been shown to activate the immune system, which eliminates damaged cells and facilitates tissue regeneration. Conversely, persistent senescence due to ageing or other persistent stimuli is detrimental.^27^ Senescent cells exhibit genomic and subcellular signalling pathway alterations in anti‐apoptotic pathways (SCAPs), including B‐cell lymphoma 2 family inhibitors (BCL‐ 2, BCL‐XL and BCL‐W), phosphoinositide 3 kinase (PI3K)/AKT, p53/p21Cip1/serpin pathways, dependence receptors/tyrosine kinases, hypoxia‐induced factor 1 alpha (HIF‐1α) and heat shock protein (HSP)90. Such cells are resistant to apoptosis and have been used to study biological regeneration and degeneration.^32^, ^33^

Despite the identification of pathways that mediate senescent cell cycle arrest, biomarkers of senescent cells have not been identified. There is no commonly used biomarker that is specific or universal for all senescent cell types, making the detection of senescent cells challenging.^10^ The most commonly used biomarker for senescence is senescence‐associated β‐galactosidase (SA‐β‐gal) activity. In normal cells, it is mainly found in lysosomes (approximately pH 4) and accumulates in senescent cells at a higher pH (pH 6).^34^, ^35^ Other markers, such as p21, p53, p16 and γH2AX, are associated with the DNA damage response and a shift in optimum pH for SA‐β‐gal.^36^ However, none of these markers are specific or universal for all senescent cell types, with ample evidence that senescent cells express most of these markers.^10^

Senescent cells secrete hundreds of factors termed the SASP, which include inflammatory and immune‐modulatory cytokines and chemokines.^37^, ^38^, ^39^ The SASP can be beneficial or detrimental within tissue micro‐environments. The SASP is protective by provoking immune surveillance of senescent cells, resulting in elimination.^40^, ^41^, ^42^ For example, ‘classically activated’ M1 macrophages have a natural role in pathogen defence and tumour protection, whereas ‘alternatively activated’ M2 macrophages can promote angiogenesis and tissue remodelling. In a fibrosis‐associated liver cancer model, SASP facilitated macrophage polarization to an M1 state, capable of attacking senescent cells in culture, contributing to an antitumor micro‐environment.^42^ In contrast, the persistent presence of SASP factors is harmful and promotes tumorigenesis, inducing chronic inflammation. For example, co‐culture of senescent cells with young cells caused premature cellular senescence of the young cells via SASP factors and gap junction‐mediated cell–cell contact.^43^, ^44^ Indeed, the composition of the SASP is highly cell‐specific and varies substantially in the same cell type with dependence upon the type of senescence and stimulus origin. Several common factors of the SASP include tumour necrosis factor α (TNF‐α), interleukin (IL)‐1, IL‐6 and matrix metalloproteinase (MMP) 13, all of which are known mediators of OP.^18^ Many studies have shown that the SASP is typically connected to the DNA damage response (DDR), possibly independent of cell cycle arrest,^10^, ^45^, ^46^ although the signalling pathway involved is unclear. Therefore, a further understanding of SASP regulation is essential to senescence research.

Interestingly, it has been reported that senescent cells display both beneficial and detrimental effects on tissues that rely on their SASP. Transiently increased senescent cells secrete SASP factors that activate the immune system and clear damaged cells, playing beneficial roles in wound healing,^47^ embryogenesis,^48^, ^49^, ^50^ cancer prevention,^51^ and ageing tissue regeneration.^40^ For example, senescent cells release MMPs to limit liver injury fibrosis and skin injury, which benefit wound healing.^40^, ^52^ Likewise, IL‐6 secreted by senescent cells promotes skeletal muscle repair following injury in vivo.^53^ Detrimental effects may contribute to age‐associated diseases, including diabetes, hypertension, and atherosclerosis.^54^, ^55^, ^56^, ^57^ Further, injection of senescent preadipocytes (representing <1% of cells) results in widespread physical dysfunction in young mice.^58^ p16INK4a is highly expressed in insulin‐producing β cells of the pancreas, with loss of p16INK4a associated with enhanced β cell replication in ageing mice. Ageing individuals are at increased risk for type 2 diabetes because of higher levels of senescent β cells.^59^ Further, removal of senescent cells attenuates the ageing phenotype in human and mouse cells.^31^ Inducible depletion of p16INK4a cells in the BubR1 progeroid mouse model delayed tissue dysfunction of adipose, skeletal muscle, and eye tissue.^31^

## 3 SENESCENCE IN BONE CELLS

**FIGURE 1:**
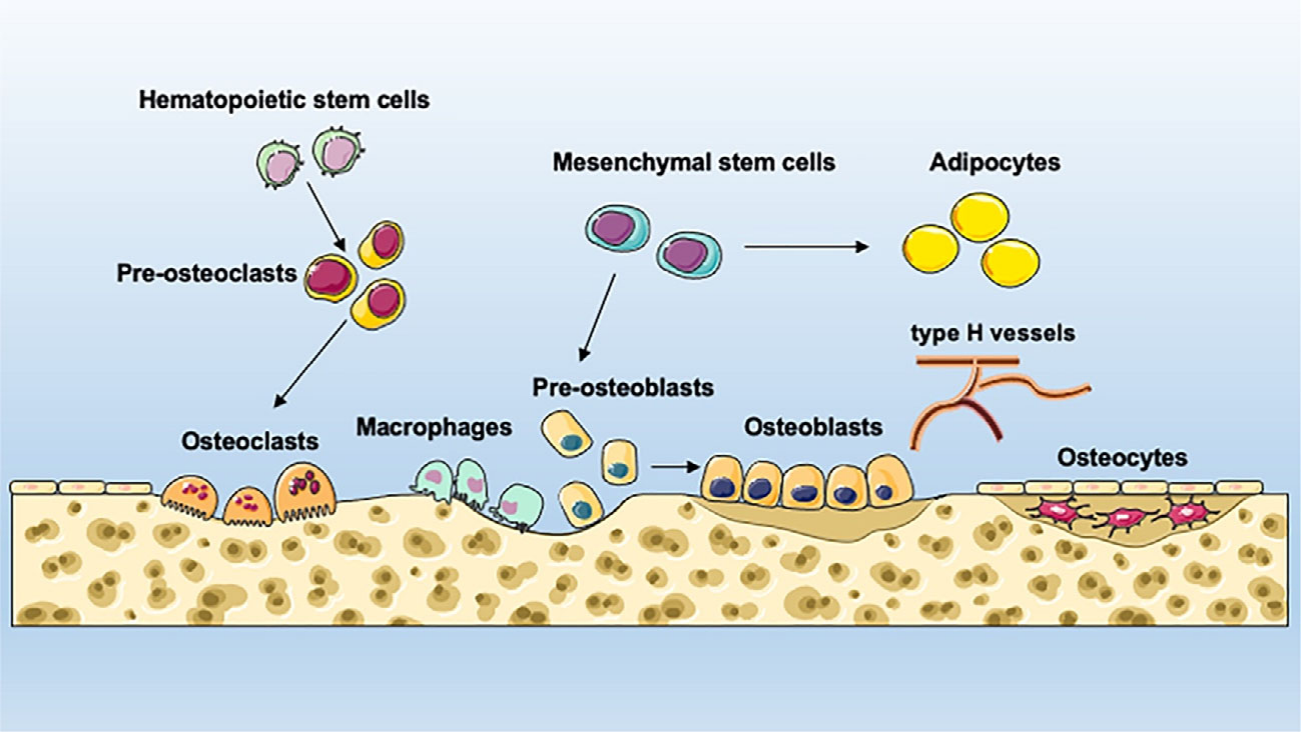
Overview of bone marrow environment. Bone is a metabolically active tissue involved in physiological processes of locomotion, support and protection of soft tissues, calcium and phosphate storage, and harbouring of bone marrow. Bone is constituted by various types of cells undergoing continuous remodelling. The maintenance of bone metabolism includes bone formation by osteoblasts and resorption by osteoclasts. Osteoclasts are terminally differentiated multinucleated cells that are derived from mononuclear cells (macrophages) of the haematopoietic stem cell lineage. Mesenchymal stem cells are progenitors of osteoblasts and adipocytes. Type H vessel formation couples osteogenesis with angiogenesis in the bone marrow and promotes bone formation. Osteocytes are terminally differentiated osteoblasts embedded in bone matrix cells.

Bone is a metabolically active tissue involved in the physiological processes of locomotion, providing structural support, calcium and phosphate regulation and storage, as well as a location for the bone marrow^60^ (Figure 1). Bone is composed of various cell types that undergo continuous remodelling.^60^ The maintenance of bone metabolism includes bone formation by osteoblasts and resorption by osteoclasts.^60^ Osteoclasts are terminally differentiated multinucleated cells expressing receptor activator of nuclear factor kappa‐B (NF‐κB) ligand (RANKL) and macrophage colony‐stimulating factor (M‐CSF).^61^ Osteoclasts are derived from mononuclear cells (macrophages) of the haematopoietic stem cell lineage. Mature osteoclasts, multinuclear cells generated from the fusion of tartrate‐resistant acid phosphatase‐positive (TRAP^+^) mononuclear cells, are primarily responsible for bone matrix resorption.^61^, ^62^ TRAP^+^ mononuclear cells are the major source of platelet‐derived growth factor (PDGF)‐BB that induces type H vessel (CD31^hi^Emcn^hi^) formation, coupling osteogenesis with angiogenesis in the bone marrow, which promotes bone formation.^63^, ^64^, ^65^ Bone marrow stem cells (BMSCs) are progenitors of osteoblasts and adipocytes, which are modulated by Wnt signalling pathways and bone morphogenetic proteins (BMPs).^66^ Osteocytes are terminally differentiated osteoblasts embedded in the bone matrix. They are closely associated with other cells and are a major source of sclerostin (SOST) and RANKL, which regulate osteoblast formation and osteoclast formation, respectively.^67^, ^68^ Osteocytes also deposit minerals and form the collagen‐enriched bone matrix that converts mechanical stimuli into biochemical signals.^69^ With physiological conditions, a balance between bone resorption and formation exists naturally. When this balance is broken, increasing osteoclast activity decreases osteoblast function, ultimately resulting in bone loss and OP.^70^

### 3.1 Senescence in bone marrow mesenchymal stem cells

Bone tissue is a metabolically active connective tissue undergoing constant remodelling.^60^ The two complementary processes: formation of new bone by osteoblasts and the resorption of old and damaged tissues by osteoclasts maintains bone homeostasis.^60^ BMSCs play a crucial role in dynamic bone balance by differentiation into osteoblasts and recruitment to sites of bone resorption, mediated by the transforming growth factor‐β (TGF‐β) 1 signalling pathway.^71^, ^72^ Mesenchymal stem cells (MSCs) also maintain haematopoietic stem cells (HSC) function.^73^ After serial passage, MSCs have a reduced capacity to differentiate into osteogenic lineages and downregulate alkaline phosphatase (ALP), collagen type 1 (Col I), Runx2 and steric.^74^, ^75^, ^76^, ^77^ These MSCs upregulate adipogenesis CEBPα, CEBPβ, CEBPγ, and peroxisome proliferator‐activated receptor (PPAR)γ.^78^, ^79^ This phenotype reinforces a pro‐adipogenic micro‐environment found during ageing.^80^ However, irradiation‐mediated senescence of BMSCs decreases levels of both osteogenic differentiation and adipogenic differentiation,^81^ which results in eventual bone loss.^81^

Increased energy is needed for stem cell differentiation, which is dependent on increased anabolism, protein turnover, lysosome‐mediated degradation, and autophagy. Autophagy is a tightly orchestrated process that sequesters misfolded proteins, damaged or aged organelles, and mutated proteins into double‐membrane vesicles.^82^ Autophagy is a suitable energy‐refuelling process required for cell differentiation. Cellular senescence has been reported to restrict autophagy activation of BMSCs, consistent with similar results for stem cells.^83^ Further, impaired osteogenic differentiation of human (h)BMSCs is attributed to defective autophagy in response to cellular senescence. Rapamycin (RAP) is a specific inhibitor of mammalian target of rapamycin (mTOR), which increases autophagy.^84^ 3‐MA is a commonly used inhibitor of autophagosome formation, which decreases autophagy.^85^ RAP elevated autophagy levels enhance the osteogenic differentiation of hBMSCs, while 3‐MA decreases the osteogenic differentiation of senescent hBMSCs.^86^ Mitophagy, the selective degradation of mitochondria through autophagy, maintains cell and mitochondrial homeostasis by specifically degrading damaged mitochondria.^87^ Mounting evidence has shown that mitophagy activation inhibits the senescence of BMSCs,^88^ inhibits adipogenic differentiation, and facilitates osteogenic differentiation of senescent BMSCs.^89^

**FIGURE 2:**
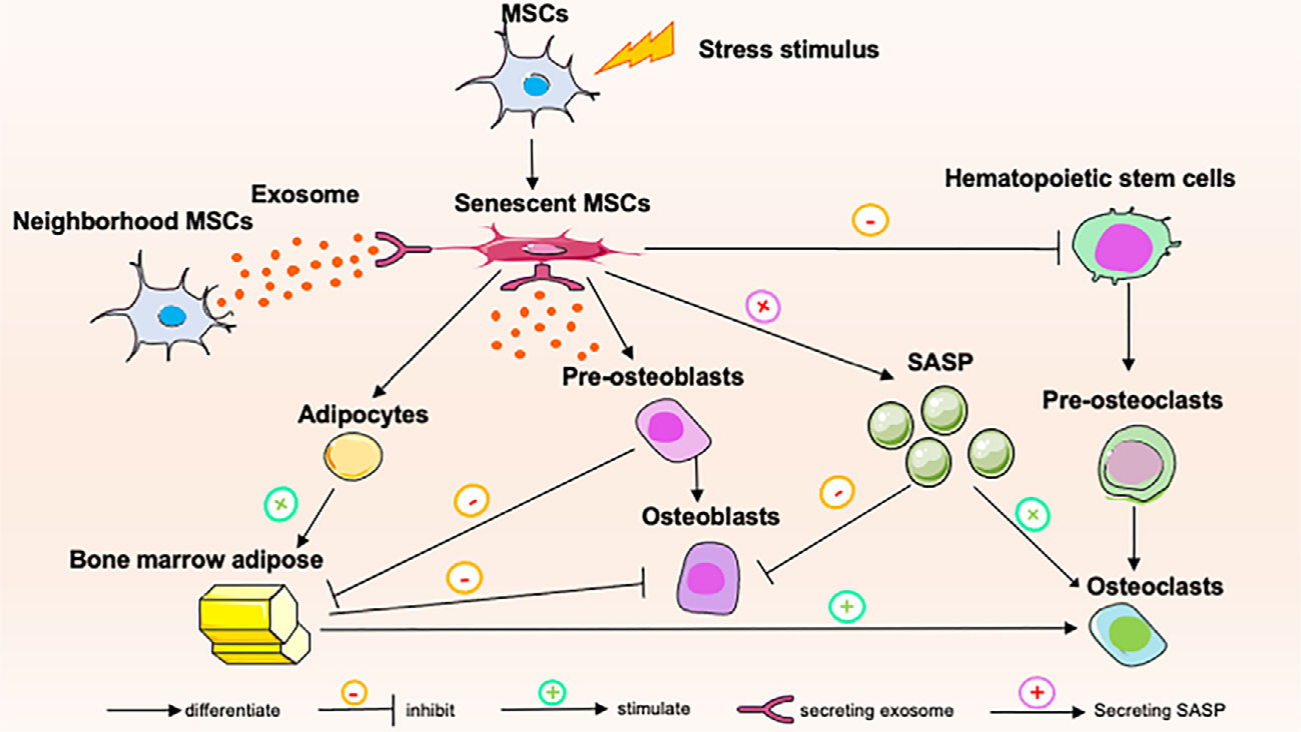
Senescence in bone marrow mesenchymal stem cells. Cellular senescence in MSCs during bone remodelling. MSCs become senescent in response to stress stimulus. Osteoadipogenic transdifferentiation in senescent MSCs has been found to increase BMAT content. BMAT exerts detrimental effects on osteoblastogenesis and positively regulates osteoclastogenesis. In addition, senescent MSCs have been reported to suppress osteoblastogenesis and stimulate osteoclastogenesis by secreting SASP. Senescent MSCs are unable to maintain haematopoietic stem cells in bone marrow. Another mechanism of senescent MSCs in osteoporosis is the negative impact of derived exosomes on healthy neighbouring cells. BMAT, bone marrow adipose tissue; MSC, mesenchymal stem cell; SASP, senescence‐associated secretory phenotype.

Furthermore, senescent BMSCs regulate bone metabolism via differentiation (Figure 2). An increase in osteo‐adipogenic trans‐differentiation of senescent MSCs increases bone marrow adipose tissue (BMAT), which is considered an endocrine organ.^90^ BMAT regulates bone remodelling through both its intrinsic properties via exosomes and indirectly through regulation of haematopoiesis, with BMAT exerting detrimental effects on osteoblastogenesis. In vitro studies have shown that co‐culture with bone marrow adipocyte media dramatically impairs osteoblast proliferation and differentiation.^91^, ^92^ BMAT is also involved in osteoclastogenesis. Bone marrow adipose lineage cell‐derived RANKL causes excess osteoclast formation and bone resorption in bone loss diseases that have increased bone marrow adiposity.^93^ In mice, ageing leads to the expansion of the adipogenic potential of a stem‐cell‐like subpopulation within the bone marrow, which in turn alters haematopoiesis through excessive production of dipeptidyl peptidase‐4 (DPP4).^94^ BMAT secretes a variety of proinflammatory cytokines and adipokines that generate an inflammatory environment within bone, which aggravates ageing and metabolic‐related disease.^95^, ^96^

In addition to influence on differentiation potential, senescent MSCs have been reported to regulate bone metabolism through the SASP (Figure 2). It has been shown that BMSCs from donors of old age produce higher levels of IL‐6 (one of the most recognized SASP factors) than BMSCs from younger donors,^97^ which induces osteoclastogenesis and suppresses osteoblast differentiation.^98^ A deficiency of IL‐6 significantly enhances Runx2 and collagen type I (col1a) gene expression in osteoblasts while decreasing the expression of osteoclast‐related genes such as TRAP, MMP9, and cathepsin K.^99^ IL‐6 deficiency alleviates BMSC senescence and prevents bone loss induced by a high‐fat diet.^100^ Moreover, soluble mediators of the SASP are released into the circulation, exacerbating chronic inflammation.^101^, ^102^ However, the specific SASP factors derived from senescent MSCs are not known, and further study is required to compare whole‐transcriptome datasets from different types of senescent MSCs, in order to identify a set of genes that are differentially expressed in senescent MSCs.

Another mechanism of MSC‐mediated OP senescence is the negative impact of extracellular vehicles (EVs) on healthy neighbouring cells (Figure 2).^103^ These vesicles act as signals, triggering senescence in healthy cells or influencing the differentiation of MSCs by secreting microRNAs (miRNAs).^104^ For example, the levels of miR‐335 are increased in aged human MSCs (hMSCs), with overexpression of miR‐335 resulting in early senescence‐like alterations, abolished osteogenic differentiation potential, and enhanced development of SASP.^105^ MiR‐188, induced by ageing BMSCs, results in increased adipogenic differentiation and inhibits osteoblastic differentiation of healthy BMSCs.^106^ Moreover, miR‐31a‐5p, derived from aged MSCs, decreases osteogenesis by BMSCs and increases osteoclastogenesis.^107^, ^108^ Furthermore, antagomir‐31a‐5p administration to bone marrow prevented age‐associated bone loss, suggesting a potential therapeutic treatment for age‐related OP.^107^ To date, specific EVs and contents for MSCs involved in OP are unknown. Targeting miRNAs may reverse the senescent phenotype of MSCs, reducing the production of the SASP and preserving bone metabolism. Future studies are needed to clarify the signalling pathways of senescence cells induced by adjacent senescent MSCs.

In summary, senescent BMSCs are crucial for OP progression; thus, transplantation of young MSCs may be an effective therapy for OP.^109^, ^110^ Importantly, MSCs can be isolated from various tissues and organs of the body, e.g. adipose tissue and bone marrow. Currently, with the advanced development of single‐cell RNA sequencing (scRNAseq), we have a comprehensive transcriptomic landscape of heterogeneous MSCs at single‐cell resolution. Several studies have shown that MSCs from different tissues have obvious differences in biological function, with MSCs from the same tissue exhibiting heterogeneity after adherent culture.^111^, ^112^, ^113^, ^114^ Therefore, future investigations should focus on identification of an MSC population that is suitable for research and/or specific treatment of OP based on biological function.

### 3.2 Senescence in osteocytes

The multi‐dendritic structure of osteocytes is an essential characteristic closely related to the osteocyte physiological function of crosstalk with other cells at the bone surface.^115^, ^116^ Osteocytes comprise >90% of all bone cells, functioning in mechanical induction and providing a key role for osteocytes in bone metabolism.^117^ Mounting evidence has shown that osteocyte senescence is involved in the disruption of bone metabolism during ageing and other pathological conditions.^118^

For instance, p16 and p21 mRNA levels are increased significantly in osteocytes from 24‐month‐old mice compared to 6‐month‐old mice,^18^ which is consistent with in vitro findings. Primary osteocytes from old mice exhibit a senescence phenotype, as judged by high levels of impaired DNA damage markers that are associated with cellular senescence.^119^ The basis for osteocyte senescence during ageing may be attributed to mitochondrial dysfunction and proteostasis disturbance. Specifically, osteocytes express two autophagy marker genes, Atg7 and Map1lc3a (commonly known as LC3), which are lower in older mice, indicating autophagy dysfunction (mitophagy).^18^ Impaired mitophagy disturbs the balance between mitochondrial biogenesis and turnover, leading to the accumulation of dysfunctional, damaged mitochondria, resulting in more reactive oxygen species (ROS) generation, and senescence.^120^ Moreover, at the molecular level, it has been proposed that loss of proteostasis and mitochondrial dysfunction may contribute to age‐related bone dysfunction.^10^ Further, proteolytic activity and the rate of protein turnover decline in aged animals^121^ and in ageing humans,^122^, ^123^ which may be one mechanism of cellular senescence.^124^ Decreased autophagy of osteocytes is linked to impaired protein homeostasis or proteostasis, which may contribute to cellular senescence.^125^

At present, the relationship between oestrogen deficiency‐induced OP and osteocyte senescence is unclear. Increased expression of senescence markers and SASP components has been found in cortical bone (contains abundant osteocytes) in an ovariectomized (OVX) mouse model.^126^, ^127^, ^128^ However, another study found no indication of senescent osteocytes postmenopausal in either humans or mice.^16^ Moreover, DNA damage was found in irradiation‐mediated osteocyte senescence,^129^, ^130^ as judged by significantly increased accumulation of γH2AX in osteocytes that was accompanied by increased expression of SA‐β‐gal, p16 and p21.^58^, ^131^ These findings indicate that at least a subset of osteocytes become senescent with age and pathological conditions, which may eventually result in bone loss. However, it is not clear whether senescent osteocytes are the primary trigger for OP, with further study needed for the breeding of conditional gene knockout mice to clarify this possibility.

**FIGURE 3:**
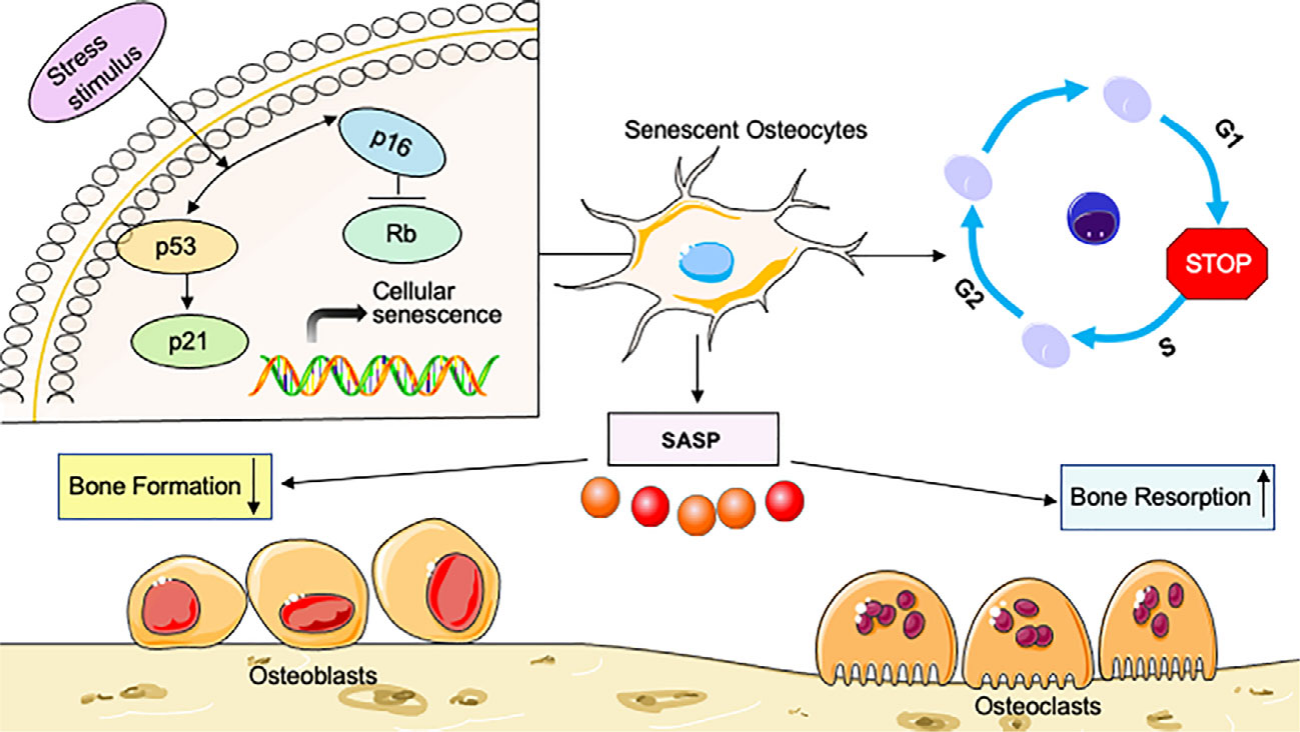
Senescence in osteocytes. A relatively small proportion of osteocytes become senescent under stress stimulus, and these cells are likely to cause an inflammatory microenvironment in bone by secreting SASP, disrupting bone formation and enhancing osteoblast function. Two main tumour suppressor‐mediated signalling pathways, p53/p21CIP1 and p16INK4a/pRB, are responsible for the growth arrest of osteocytes. SASP, senescence‐associated secretory phenotype.

Although only a relatively small proportion of osteocytes become senescent with ageing, these cells are likely to induce a bone inflammatory micro‐environment by secretion of SASP factors^18^ (Figure 3). In ageing mice, it appears that senescent osteocytes and myeloid lineage cells are the main sources for SASP factors, contributing to the development of a proinflammatory local bone micro‐environment.^18^ Previous studies reported that osteocytes regulate myeloid lineage cells by producing RANKL, which stimulates osteoclast development from myeloid progenitors.^132^ It is tempting to speculate that a subset of osteocytes is the primary trigger for cellular senescence and a SASP, leading to senescence of myeloid lineage cells and signal amplification.^18^ Certain factors that constitute the SASP, such as TNF‐α, IL‐1 and IL‐6, not only contribute to the senescence of healthy neighbouring cells and disintegration of the extracellular matrix but also stimulate bone resorption and inhibit bone formation.^133^, ^134^ For example, prematurely senescent osteocytes induced by irradiation can activate multiple SASP factors, such as TNF‐α, IL‐6, IL‐1α and MMP13.^131^ RAW264.7 cells, a commonly used osteoclast precursor cell line, when co‐cultured with osteocytes previously irradiated with 2, 4, or 8 Gy γ‐rays and treated with 25 ng/ml RANKL, exhibited osteoclastogenesis. The authors reported that compared to non‐irradiated osteocyte co‐culture, irradiated osteocytes dramatically stimulated the differentiation of osteoclast precursors as evidenced by TRAP staining, in a dose‐dependent manner.^131^ In vitro experiments suggest that secreted factors (including IL‐1α, IL‐1β, IL‐6, IL‐17 and MCP1) in senescent osteocyte medium can reduce osteo‐progenitor cell recruitment, disrupting subsequent bone formation, inhibiting osteoblast differentiation, and impairing mineralization.^134^ Moreover, senescent osteocyte‐associated factors aggravate lipopolysaccharide (LPS) inhibitory effects on osteoblast differentiation and mineralization by regulating key osteogenic and mineralization genes, such as Runx2 and Osterix.^134^ Further, both osteogenic and adipogenic differentiation of BMSCs was significantly decreased when BMSCs were co‐cultured with irradiated osteocytes. Bone areas of positive alkaline phosphatase, mineralized nodules stained with Alizarin Red S, and lipid droplets stained with Oil Red O were significantly decreased compared to controls.^135^ Interestingly, use of a 0.8 μM JAK1 inhibitor blocked SASP secretion from irradiated MLO‐Y4 cells, which negated the inhibition of BMSC differentiation.^135^

Therefore, senescent osteocytes can regulate bone metabolism through the SASP. However, the composition of the SASP derived from senescent osteocytes may differ with distinct stimuli. Further investigation is required to clarify this issue.

## 4 CELLULAR SENESCENCE IN OP

### 4.1 Cellular senescence in primary and postmenopausal OP

Oestrogen maintains bone homeostasis by balancing cell survival and death within bone.^136^ In menopausal women, approximately 20%–30% of trabecular bone and 5%–10% cortical bone^137^, ^138^ are lost.

Postmenopausal bone loss occurs in two phases. The high bone turnover phase is characterized by the increased concurrent bone formation and resorption. This initial phase lasts 3–5 years.^60^, ^139^ A deficiency of oestrogen stimulates rapid osteoclastogenesis, which induces bone resorption at the surface of trabecular bone.^60^, ^139^ Oestrogen deficiency also induces osteocyte apoptosis and the release of RANKL, which stimulates osteoclasts and upregulates SOST. SOST inhibits WNT signalling, increasing osteoblast number, and decreasing the activity of osteoblasts.^60^, ^139^ As a result, bone resorption outpaces bone formation, leading to rapid net bone loss.^60^, ^139^ Bone loss is most significant in trabecular bone, with impairment of trabecular microstructure and loss of trabecular elements.^60^, ^139^ The second phase of OP in postmenopausal women is associated with slow bone loss, which resembles senile OP that lasts 10–20 years.^60^, ^139^

Oestrogen is crucial for the survival and function of osteoblasts and osteocytes, and has the capacity to relieve senescence of osteoblasts and osteocytes.^140^ The mechano‐sensation function of osteocytes is disrupted for a long period of time in postmenopausal OP,^60^, ^139^ which may be attributed to the senescence of osteocytes.^126^, ^127^, ^128^ In OVX mice, osteocytes not only had higher percentages of p16 and β‐galactosidase^127^ but also produced more SASP components, such as MMP‐3, MMP‐13, IL‐6, IL‐8, IL‐1α and IL‐1β.^127^ Further, exogenous oestrogen supplementation inhibited osteocyte senescence and the SASP, rescuing bone loss in OVX mice.^127^ Knocking out p16 in OVX mice decreased the proportion of β‐gal‐positive osteocytes and p21 protein levels in bony tissue, with prevention of bone loss compared to WT‐OVX mice.^128^ These results indicate that oestrogen deficiency induces bone loss, partly through senescence of osteocytes. The means by which oestrogen exerts anti‐senescence effects are not fully understood, although inhibition of Usp10 may be involved. Usp10, an important deubiquitination enzyme, which maintains the stability and function of p53 by direct removal of ubiquitin molecules from p53.^126^ Treating MLO‐Y4 cells with oestrogen decreases the mRNA and protein levels of p53 and Usp10. The inhibition of Usp10 attenuates senescence in both the osteocyte cell line, MLO‐Y4, and the osteoblast cell line, MC3T3‐E1, by downregulation of p53 and p21, preventing bone loss in OVX mice.^126^

Senescent BMSCs are found in OVX mice, as judged by increased dual staining with γH2AX and another BMSC marker, leptin receptor (LepR).^141^ Moreover, elevated SA‐β‐gal‐positive cells and fewer Ki67 (cell proliferation marker)‐positive cells were observed in BMSCs from OVX mice. Senescent BMSCs from OVX mice exhibit impaired osteogenesis, as judged by lower osteogenic markers (ALP, Runx2 and osteocalcin) and fewer mineralized nodules, as judged by Alizarin Red staining.^141^ In vitro, 10^−7^ mol/L 17β‐estradiol decreased senescence and restored osteogenic differentiation of BMSCs.^141^ Oestrogen reversed BMSC senescence by modulating the SASP and JAK2/STAT3.^141^ Further, decreased SASP is associated with decreased cellular senescence of BMSCs. OVX mice treated with a JAK inhibitor (25 mg/kg, drug/body weight) every other day for a 3‐month period exhibited SASP inhibition^142^ with reduced BMSC senescence and bone loss.^141^

However, one recent study of humans and mice found that oestrogen deficiency did not alter senescence biomarker levels or SASP components in bone. Treating INK‐ATTAC mice with AP20187, eliminated p16INK4a‐senescent cells, but did not prevent bone loss after OVX.^16^ Therefore, it is unknown whether oestrogen deficiency‐induced bone loss depends on cellular senescence. Changes in the micro‐environment can influence the progression of cellular senescence, which may be explained by alterations in the level of stress during pathological processes. Senescence progression is not only influenced by oestrogen but also determined by oxidative stress.^143^ Oestrogen deficiency can induce oxidative stress and reduce antioxidant level and activity, whereas 17β‐estradiol supplementation can reduce oxidative stress by increasing antioxidant level and activity in OVX mice.^127^ Oestrogen deficiency induces ROS through downregulation of B lymphoma Mo‐MLV insertion region 1 (Bmi1),^127^, ^144^ which is a member of the polycomb family of transcriptional repressors that regulate cell cycling and senescence by downregulation of p16INK4a/Rb and p19AFR/p53 pathways.^145^ Thus, a complex relationship may exist among oestrogen, senescence, ROS and the micro‐environment. This speculation warrants further investigation.^10^

Overall, oestrogen may be essential to postmenopausal OP. However, data from the Women's Health Initiative study indicated that oestrogen replacement increases the risk for breast cancer and cardiovascular disease. The findings of that study resulted in a considerable drop in the use of oestrogen.^146^ In addition to the regulation axis centred on the hypothalamus, other endocrine factors may also be involved in the ageing process.^147^ During ageing, hormone secretion by the hypothalamic–pituitary axis is altered and feedback sensitivity is modulated, contributing to pathological conditions.^147^ For example, the stability of blood calcium level is maintained by balanced secretion of parathyroid hormone (PTH) and calcitonin (CT).^148^, ^149^ When the blood calcium levels are low, PTH levels are increased, enhancing renal tubule and small intestine uptake and absorption of calcium, which stimulates osteoclast activity. More bone calcium is decomposed and released into the blood, rapidly increasing blood calcium levels.^149^ At the same time, thyroid C cells increase CT secretion, reducing the uptake and absorption of calcium by the renal tubules and small intestine while inhibiting bone osteoclast activity such that blood calcium is fixed as bone calcium, thus reducing blood calcium levels.^148^ Specifically, short or intermittent PTH treatment can significantly increase osteoblast‐mediated bone formation, while continuous high‐dose PTH treatment stimulates greater bone resorption than bone formation, resulting in bone loss.^150^, ^151^ Further, results have linked increased PTH serum levels^152^, ^153^ and decreased levels of CT with age^154^ and possible bone loss. Moreover, oestrogen can protect against increased bone resorption induced by PTH infusion.^155^ PTH's synthetic N‐terminal teriparatide increases bone mass (with a slight increase in bone resorption) and has been approved by the FDA for clinical use.^2^, ^156^ The IGF‐1 signalling pathway plays an anabolic role in bone metabolism by increasing bone formation,^157^, ^158^ with decreased levels of IGF‐1 associated with advancing ageing and an increased risk for OP.^159^, ^160^, ^161^ Treatment of ageing animals with IGF‐I stimulates bone formation and regeneration in aged animal models.^162^ Moreover, glucose homoeostasis, which is under tight hormonal control by insulin, is dependent on a balance between glucose ingestion, utilization, and production. Advanced age is related to a redistribution of fat depots, increasing the percentage of total body fat,^163^, ^164^, ^165^, ^166^ obesity (particularly visceral fat deposits) and lipid spillover into muscle.^54^, ^167^, ^168^, ^169^, ^170^ This redistribution decreases insulin action with advancing age, placing glucose homoeostasis into disequilibrium.^147^, ^171^, ^172^ Future studies are essential to assess the relationships among cellular senescence, oestrogen, OP, and the hypothalamic–pituitary axis.

### 4.2 Cellular senescence in primary and senile OP

Senile OP is a human, worldwide metabolic bone disorder with a high incidence that is characterized by the loss of both cortical and trabecular bone.^60^, ^173^ Trabecular bone resorption and formation are reduced with increased age.^60^, ^173^ Moreover, maintenance of bone homeostasis is due to both local factors (such as growth factors and cytokines) and systemic factors (such as calcitonin).^60^, ^173^ However, during ageing, the loss of cortical bone is most significant, due to enhanced porosity, increased resorption and incompletely closed osteon.^60^, ^174^

Osteocyte dysfunctions and osteoblast dysfunction are considered the primary causes of senile OP.^60^, ^173^ The key senescence marker, *p16INK4a*, is significantly increased in osteocytes of aged male and female mice.^18^ Further, SASP genes are significantly upregulated in old osteocytes. Consistent with the data from mice, bone biopsies (osteocyte‐enriched samples) from elderly women have significantly elevated mRNA expression of p16INK4a and p21, as well as several SASP factors, when compared to biopsies from younger women.^18^ Another study showed that osterix‐expressing osteo‐progenitors from old mice exhibit several markers of senescence (e.g., *γ*H2AX and p53) as well as a SASP.^175^ Large numbers of osteoblasts in old mice exhibit cellular senescence markers, including β‐galactosidase, p16INK4a, DNA methylation marker 5‐methylcytosine (5mC), and oxidative damage marker 8‐hydroxydeoxyguanosine (8‐OHdG), when compared to controls.^176^ As such, osteoblasts and osteocytes derived from older individuals exhibit senescence and a SASP.^177^

Senescent BMSCs, including those that have stem‐cell‐like properties, alter the differentiation of osteogenic and adipogenic cells and contribute to senile OP.^15^, ^178^, ^179^ Further, mitophagy is markedly reduced during normal BMSCs ageing, which facilitates adipogenic differentiation at the expense of osteogenic differentiation.^180^ Decreased levels of autophagy, caused by ageing, are related to impaired BMSC osteogenic capacity during senile OP.^86^, ^89^ LepR is a marker for bone BMSCs. Approximately 0.3% of bone marrow cells are LepR^+^ ^73^ and these cells are a major source of osteoblasts and adipocytes.^181^ In human BMSCs, LepR expression is upregulated with ageing.^182^ With age, a large proportion of LepR^+^ cells become senescent, as judged by high levels of p16 in murine femurs.^183^

The immune system is crucial to understanding bone homeostasis and bone pathology. Farr et al. found that not only osteocytes but also myeloid lineage cells, particularly macrophages (expressing *p16INK4a*), are senescent and secrete SASP factors.^18^ Polarization of macrophages toward the M1 phenotype and cellular senescence are induced by *p16INK4a*.^13^, ^184^ Li et al. reported that proinflammatory and senescent neutrophils and macrophages accumulate in the bone marrow and induce skeletal ageing in rats and mice by secreting abundant quantities of grancalcin, which lowers bone turnover and increases bone marrow fat.^185^ Mechanistically, grancalcin was found to bind and inhibit plexin‐b2 receptor signalling by BMSCs, decreasing osteogenesis and stimulating adipogenesis of BMSCs.^185^ In contrast, genetic deletion of grancalcin in neutrophils and macrophages or the use of grancalcin‐neutralizing antibodies delayed skeletal ageing.^185^ Taken together, these results suggest that senescent immune cells are potential targets for age‐related OP.

The results above suggest that an accumulation of senescent bone cells and a SASP may result in primary OP and it is therefore possible that the elimination of senescent cells will protect from age‐related bone loss. Both genetic and pharmacological approaches have been used to eliminate senescent cells. AP20187 treatment, which eliminates p16+ cells in old *INK‐ATTAC* mice, reduces age‐related trabecular bone loss of the spine,^31^, ^142^, ^186^ increases cortical bone mass of the femur and improves bone strength at both sites.^17^ Pretreatment of whole mouse bone marrow with senescent osteocyte‐conditioned medium increased osteoclast differentiation, indicating that SASP factors secreted from senescent cells promote osteoclast progenitor survival.^17^ Furthermore, old mice that received either 4 months of senolytic administration (which eliminates senescent cells) or a 2‐month JAK inhibitor (which blocked the proinflammatory secretome of senescent cells), ‘senomorphic approach’^142^, ^186^ showed improved bone microarchitecture and strength compared to old male WT mice.^17^ Further, aged mice treated with tetramethylpyrazine (TMP), the bioactive component extracted from *Ligusticum wallichii Franchat* (Chuanxiong), had increased trabecular bone microarchitecture. A potential explanation for this phenomenon is that TMP eliminates the senescent phenotype of LepR^+^ bone marrow stem/progenitor cells.^183^ Likewise, the administration of senomorphic drugs and ruxolitinib to old mice improved physical function^142^ and increased lifespan.^58^ These findings suggest that specific targeting of senescent MSCs or osteocytes may provide a novel therapeutic strategy by which to not only prevent bone loss but also alleviate frailty.

In the clinic, ageing cortical bone loss is more significant than trabecular bone loss, indicating that two different mechanisms underlie bone loss in these two compartments. With ageing, both the number of osteoclasts and the degree of bone resorption decrease in trabecular bone, while osteoclastogenesis increases in cortical bone.^187^, ^188^ Effective killing of senescent osteocytes in the bones of aged mice has been shown to reduce *IL‐1a* and *Tnfsf11* mRNA,^189^ decrease osteoclast number on the endo‐cortical surface, and increase cortical bone mass. This may be due to Tnfsf11‐ and SASP‐induced RANKL production by osteocytes of cortical bone, which stimulates osteoclastogenesis and bone loss.^190^ Another study confirmed this possibility. Treating p16‐3MR mice with ganciclovir eliminated osteoclast progenitors but did not prevent cortical bone loss in aged mice.^191^ Further study is needed to determine the life span of osteocytes in trabecular and cortical bone.^189^

### 4.3 Secondary OP senescence and GC‐induced OP

GCs are an effective treatment for a wide range of inflammatory diseases, such as rheumatoid arthritis (RA) and ankylosing spondylitis (AS).^192^, ^193^ However, clinical experience has shown that in the first 3–6 months of treatment with daily dosages ranging from 2.5 to 7.5 mg, there is an increase in bone fragility and subsequent fracture, which results in OP, extensive medical issues, and socioeconomic burden.^60^ GIOP is a secondary form of OP,^194^ with an unknown mechanism of action. GCs have detrimental effects on bone cells. A high dose of GCs negatively regulates the osteogenic differentiation of MSCs. Exogenous GCs induce apoptosis of osteoblasts and osteocytes. Apoptotic osteocytes are the main source of SOST and RANKL, negatively regulating bone formation and positively regulating bone resorption.^60^, ^68^, ^195^

Recently, cellular senescence has been shown to play a role in various cell types (e.g. MSCs) in response to GC treatment.^196^ For example, in young mice Nestin‐expressing (Nestin^+^ cells) in postnatal bones are primarily of endothelial and osteoblast lineages,^197^ known to undergo GC mediated senescence.^198^, ^199^ Further, decreased angiogenesis is responsible for rapid bone loss in paediatric GIOP. GC use induces endothelial cell senescence in the metaphysis of long bone resulting in bone loss, while blockade of endothelial senescence prevents bone loss.^200^ Moreover, ANG, a ribonuclease that is secreted by osteoclasts, is essential for senescence protection of neighbouring blood vessels through an ANG/PLXNB2‐rRNA transcription signalling pathway.^200^ GC treatment induces blood vessel cell senescence by suppressing the formation of ANG‐expressing osteoclasts in the metaphysis, which is accompanied by reduced angiogenesis‐coupled osteogenesis.^10^, ^200^

**FIGURE 4:**
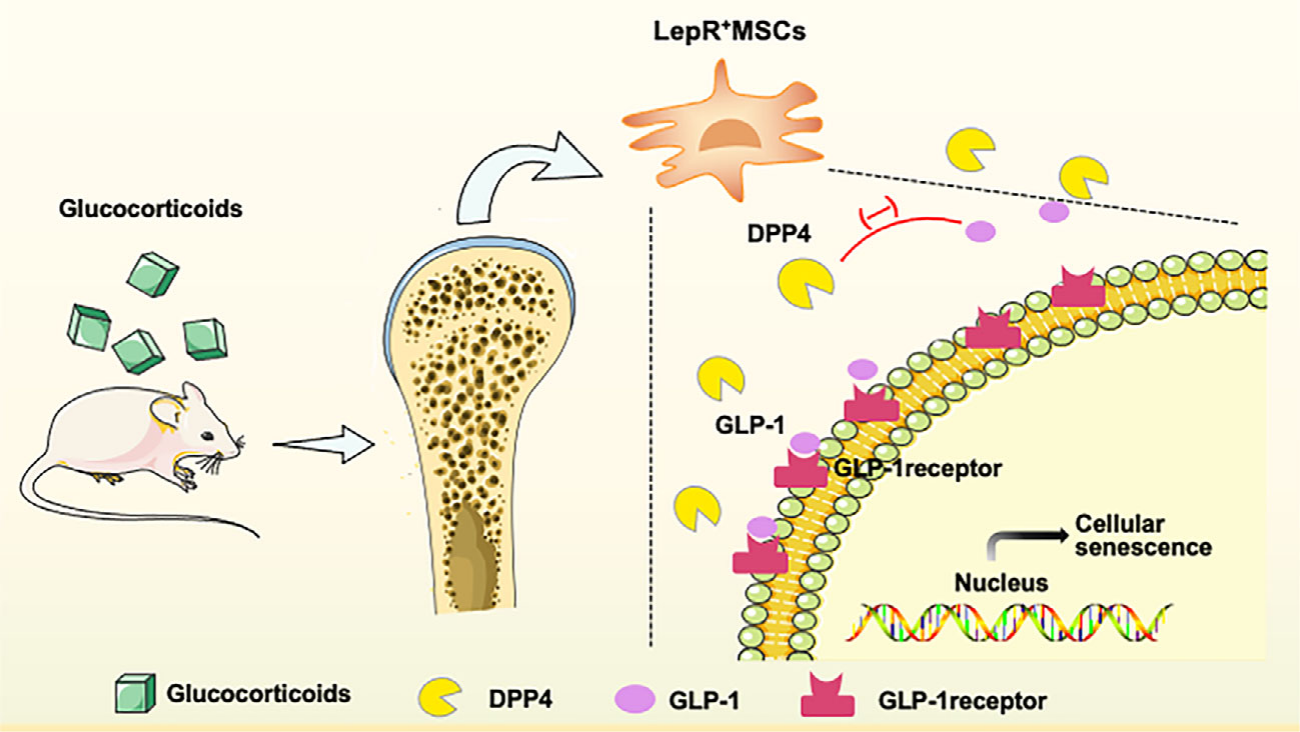
Glucocorticoids induce (LepR^+^ MSCs) senescence through the DPP4/GLP‐1 transcription signalling pathway. DPP4, dipeptidyl peptidase‐4; GLP‐1, glucagon‐like peptide 1; LepR^+^ MSCs, leptin receptor‐positive mesenchymal stem cells.

In addition to young mice, LepR^+^ MSCs of adult mice are also susceptible to GC treatment.^12^ Flow cytometry demonstrated LepR^+^ cells to exhibit a senescent phenotype with increased p16INK4a, p53 and p21 expression, confirming LepR^+^ cellular senescence in GC‐treated bone marrow.^12^ Clearance of senescent cells by dasatinib (D) + quercetin (Q) rescues GC‐induced bone loss.^12^ DPP4, a membrane glycoprotein with exopeptidase activity, was recently reported to play an important role in the inflammatory macrophage profile associated with type 2 diabetes, obesity, and OP.^12^, ^201^ DPP4 selectively cleaves alanine and proline from polypeptide substrates that result in substrate degradation of glucagon‐like peptide 1 (GLP‐1) and gastric inhibitory polypeptide (GIP).^202^ GC treatment upregulates DPP4 and downregulates GLP‐1, resulting in LepR^+^ MSC senescence and disrupted bone osteogenesis and angiogenesis.^12^ These observations identify cellular senescence as a new means by which GCs exert deleterious effects on bone microarchitecture, suggesting the DPP4/GLP‐1 axis to be a regulator of GC‐induced LepR^+^ cell senescence in adult mice. In the future, it is important to determine whether a decline or loss of DPP4/GLP‐1 axis signalling in young or adult bone is due to the cellular senescence associated with advanced ageing and other pathological conditions (Figure 4).

### 4.4 Senescence and inflammatory bone loss

Patients with inflammatory diseases are at high risk for bone loss.^203^ For example, Hauser et al. reported that patients suffering from rheumatoid arthritis had an overall OP prevalence of 26.5%, which was significantly higher than the prevalence of OP in a gender‐ and age‐matched control cohort.^204^ LPS, a gram‐negative bacterial outer membrane component, induces critical inflammatory factors including TNF‐α, IL‐1 and IL‐6 that activate inflammation‐induced bone resorption.^203^, ^205^ These factors work with a multitude of cells that activate pre‐osteoclasts, increasing the number of mature osteoclasts and the area of eroded surface through autocrine, paracrine, and endocrine mechanisms.^205^ These factors also inhibit osteoblast function, decreasing bone formation.^203^, ^205^

Repeated LPS exposure can induce senescence in microglia,^206^ dental pulp^207^ and pulmonary epithelial cells.^208^ Senescent osteocytes induced by LPS are responsible for inflammatory bone loss.^209^ LPS administration induces osteocyte senescence, as demonstrated by a significant increase in the expression of p16INK4a and p21 in alveolar bone, accompanied by increased γH2AX immunoreactivity. LPS exposure also enhances the production of proinflammatory factors, including intercellular cell adhesion molecule‐1 (ICAM1), IL‐6, monocyte chemoattractant protein‐1 (MCP1), MMP12 and MMP13.^209^ In an ex vivo model that mimics the in vivo situation, tissues and cells were morphologically positioned within the normal extracellular matrix.^210^ With exposure of alveolar bone to LPS, *p53 was significantly increased b*ut not *p16INK4* or *p21*. Increased levels of IL‐1α, IL‐6 and TNF‐α were observed as well.^209^ These data suggest that persistent LPS exposure induces senescence of alveolar‐derived, osteocyte‐like cells by promotion of DNA damage via p53 activation.^209^ Moreover, LPS has been shown to increase the production of IL‐1α, IL‐6 and TNF‐α through p53‐dependent activation of human gingival fibroblasts.^211^ In conclusion, LPS‐triggered activation of p53, rather than p16, induces senescent osteocytes to secrete SASP factors in vivo, resulting in DNA damage.^209^, ^212^

## 5 TARGETING CELLULAR SENESCENCE AS A PROMISING THERAPEUTIC STRATEGY FOR OP

Current treatment options for OP either suppress bone resorption or stimulate bone formation, but have limited benefit. Recent evidence has demonstrated a link between senescence and OP, providing for potentially exciting strategies by which to prevent and treat OP. Possible therapeutic treatments include senescent cell‐targeting, SASP‐targeting therapies, gene therapy to rejuvenate stem cells, and treatment with traditional Chinese medical herbs. In what follows, we describe the potential benefits of each of these and the mechanistic basis for each strategy.

### 5.1 Selective elimination of senescent cells

Resistance to apoptosis is a significant hallmark of senescent cells. To achieve this protection, senescent cells upregulate several SCAPs. Therefore, targeting these networks directly and eliminating these cells may prevent the initiation and progression of OP.^186^, ^213^

ABT263, a specific inhibitor of the anti‐apoptotic proteins, BCL‐2 and BCL‐x, counteracts their anti‐apoptotic function and has been widely used to eliminate senescent cells.^58^, ^133^, ^214^, ^215^ Oral administration of ABT263 to either sub‐lethally irradiated or normally aged mice effectively depletes senescent cells, as well as removes senescent muscle stem cells.^216^ Treating 24‐month‐old female mice with ABT263 for 5 days reduced levels of the DNA damage marker, H2AX, and the senescence markers, p16 and GATA4, in osteocyte‐enriched bone.^189^, ^217^ Surprisingly, a negative effect was observed in vivo for 24‐month‐old male and female mice treated with ABT263 for 2 weeks, with mice exhibiting trabecular bone loss in the proximal tibia, which contributed to impaired osteo‐progenitor function.^218^ Further experiments are required to clarify the potential use of ABT263 for treatment of OP. The haematological toxicity of general inhibitors of BCL‐2 has been evaluated for flavone, fisetin and the BCL‐X_L_ inhibitors A1331852 and A1155463.^133^, ^219^ Fisetin exerts anti‐inflammatory effects, promotes osteoblast differentiation, promotes osteogenesis,^220^, ^221^ suppresses osteoclast activity,^221^ and antagonizes OP.^220^, ^221^ Another flavonoid, fenofibrate, stimulates the differentiation of osteoblasts into osteogenic precursor cells through the induction of PPARα‐mediated BMP2 expression.^222^

Targeting senescence‐specific pathways for depletion of senescent cells do appear to be effective as an OP treatment. Regulation of p53 is at the posttranscriptional and in particular the protein stability levels. This regulation is primarily controlled by the MDM2 E3 ubiquitin ligase that poly‐ubiquitinates and degrades p53.^223^ Inhibition of MDM2 blocks the interaction between MDM2 and p53, which elevates p53 and p21 expression.^224^ Transfection of hMSCs with an MDM2 overexpression plasmid successfully reduced the transcription and protein levels of p53 and increased osteogenic differentiation of the MDM2 plasmid‐treated hMSCs compared to untreated control and empty vector‐treated cells.^225^ These results indicate that MDM2 may act as an antagonizer of OP by inducing p53 degradation.

HSP90 plays an important role in various cell functions, including protein folding and stabilization, proteasomal degradation and the cellular stress response.^226^ Mice treated with HSP90 inhibitors exhibit decreased p16 and delayed onset of several age‐related symptoms and increased lifespan.^227^ It is still unclear whether HSP90 inhibitors are a potential treatment for OP. Results regarding the role of HSP90 in osteoclastogenesis are controversial. SNX‐2112, an HSP90 inhibitor, suppresses osteoclast formation in vivo and in vitro.^228^ Other studies have demonstrated that inhibition of HSP90 enhances osteoclastogenesis by activating Src kinase, which is a non‐receptor tyrosine kinase that induces resistance to apoptosis.^229^, ^230^, ^231^, ^232^ The role of HSP90 in osteoblast‐mediated bone formation is unclear. One study reported that blockade of HSP90 attenuated GC‐induced osteoblastogenesis and OP.^233^ In vitro, treatment with 17‐AAG, an HSP90 inhibitor, of C3H10T1/2 and PCOB cells, stimulates osteoblastic differentiation.^233^ These data are consistent with the finding that administration of 17‐AAG to mice promotes osteoblastogenesis rather than bone resorption, which increases bone mass during bone remodelling.^233^

Collectively, targeting senescent cells with natural products or other compounds appears to effectively alleviate OP. However, there are potential barriers to this form of treatment, including off‐target effects, exhaustion of stem cells and failure to efficiently induce apoptosis in senescent cells. More precise targeting of specific senescent cells is needed to overcome these barriers.^133^, ^234^ Immune system function declines with age, disrupting the clearance of senescent cells.^235^ Remodelling of the immune system may provide a more efficient means by which to decrease the number of senescent cells, reducing OP progression.

### 5.2 Rejuvenation of stem cells by gene therapy

Stem cell exhaustion can not only induce cellular senescence, but also cause a variety of diseases related to ageing, including OP. Therefore, stem cells may be a potential target for the prevention of OP.^236^ Further, factors that rejuvenate stem cells can prevent stem cell senescence and OP in mice.

Special AT‐rich binding protein 2 (SATB2) plays a critical role in site‐specific properties of BMSCs (stemness, anti‐ageing capacity and osteoblastic differentiation) by upregulating the activity of other DNA‐binding proteins (e.g., nuclear matrix proteins) that orchestrate chromatin organization and remodelling.^237^ Overexpression of SATB2 rejuvenates senescent BMSCs and promotes the osteogenic differentiation of these cells. Transplantation of BMSCs rejuvenated by SATB2 overexpression prevents oestrogen deficiency‐related alveolar bone loss.^238^ Alpha‐ketoglutarate (αKG), a crucial intermediate of the tricarboxylic acid cycle located between succinyl‐CoA and isocitrate, was recently reported to have anti‐ageing effects.^239^ Administration of αKG protects old mice from OP, decreases cellular senescence, and rejuvenates aged MSCs.^240^ αKG reduces overall H3K9me3 and H3K27me3 levels, which are two critical histone modifications that are closely related to cell senescence and organismal ageing.^241^, ^242^ Histone lysine demethylase 4B (KDM4B) is an H3K9me3 demethylase. Knocking out KDM4B impairs MSC self‐renewal and promotes MSC exhaustion, accelerating bone loss and marrow adiposity.^236^ Activation of KDM4B in MSCs may be an epigenetic rejuvenation strategy for the prevention or treatment of skeletal ageing.^236^ LRRc17, a vital orthotropic factor for bone metabolism,^243^ increases with age.^244^ Overexpression of LRRc17 accelerates the senescence of young mouse‐derived BMSCs, favouring adipogenic differentiation of MSCs. Knockdown of LRRc17 not only restored the morphology of mitochondria but also effectively improved mitophagy, alleviating BMSC senescence during H_2_O_2_ treatment.^244^ Transplantation of BMSCs, in which LRRc17 was knocked down, alleviated OVX‐induced bone loss.^244^ Mitochondrial dynamics play a critical role in cellular senescence, with mitochondrial impairment a prominent risk factor for bone metabolic disease.^245^ Among these, the mitochondrial deacetylase, sirtuin 3 (Sirt3), localized to the mitochondria, has been reported to inhibit mitochondrial apoptosis.^246^, ^247^ Sirt3‐mediated mitochondrial homeostasis may rejuvenate the senescence of stem cells.^248^, ^249^ In the SAMP6 mouse model of senile OP, injection of Sirt3‐Flag for 4 weeks not only reversed BMSC senescence but also promoted the secretion of ALP and reduced the secretion of TRAP5b, a bone resorption marker, indicating that Sirt3 acts as an inhibitor of OP.^89^ Collectively, targeting stem cell rejuvenation may be a potential therapeutic strategy for OP. The identification of gero‐protective factors and the appropriate targeting of such by gene therapy remains a challenge.

### 5.3 SASP inhibition

Another therapeutic approach to OP is to target specific factors associated with the SASP of senescent cells. Components of SASP (e.g., proinflammatory cytokines, chemokines and growth factors), when targeted, may prevent bone dysfunction. These components could be blocked with TNF‐α inhibitors,^250^ IL‐1 receptor antagonists,^251^ or IL‐6 antagonists.^252^ These drugs have effectively improved BMD for inflammation‐associated disease. Unfortunately, it is still unclear whether these drugs prevent the progression of OP in the clinic. IL‐17 may be a potential target for OP. IL‐17 neutralizing antibodies have prevented bone loss and senescence of the immune system in a murine OVX model.^253^, ^254^ Deletion of the principal IL‐17 receptor protects mice from OVX‐induced bone loss.^255^ To date, no clinical trials have been performed to evaluate the efficacy of the IL‐17 antibody, secukinumab, in patients with OP. It is important that new technologies identify the specific SASP of OP so that effective drug therapy can be developed. This approach to treatment will require continual, possibly lifelong therapy to combat SASP. This is a distinct disadvantage and makes this approach difficult to translate into the clinic.

### 5.4 Traditional Chinese medical herbs as possible cellular senescence‐modulating therapies

Here are many advantages to traditional Chinese medical herbs, including lower cost, fewer side effects, and better feasibility for long‐term application. Recently, some Chinese herbs have been shown to improve bone quality via regulation of cellular senescence. TMP, extracted from the Chinese herb Chuanxion, enhanced MSC viability and delayed the senescence of MSCs by suppressing the activity of NF‐κB signalling and reducing the levels of the proinflammatory factors, TNF‐α and IL‐1β.^256^ Local delivery of TMP eliminated senescent LepR+ MSCs by epigenetically modulating Ezh2‐H3K27me3, protecting trabecular bone mass in aged mice.^183^

Angelica polysaccharide (ASP), an acetone extracted polysaccharide from Chinese angelica, has various benefits, including antioxidant, antitumor, haematopoietic regulatory, immunomodulatory and radiation protective effects.^257^ ASP promotes MSC proliferation and osteoblast differentiation by enhancing the levels of Runx2, OCN, ALP and BMP‐2 protein.^258^ In vivo results confirmed that ASP prevents OVX‐induced OP by promoting bone formation in rats.^258^

Further research is required to explore the key components of traditional Chinese medical herbs that enhance the proliferation and attenuate senescence of bone cells, so that those components can be evaluated as clinical OP treatments.

## 6 CLINICAL TRANSLATION OF CELLULAR SENESCENCE TARGETING

As summarized herein, senescent cells and the SASP are central to OP, with targeting or elimination of each a possible therapeutic approach for the treatment of OP. However, translation of this approach into clinical use is a challenge. For example, administration of the drug early in life to prevent ageing seems unlikely. Further, the time required for clinical trials would be prohibitive, and long‐term use of such drugs may have side effects. Two clinical studies have begun to test the efficiency of senescent cell elimination for disease treatment in humans. One study (https://clinicaltrials.gov/ct2/show/NCT02848131) evaluated combination therapy of dasatinib (D) and quercetin (Q). Oral intake of 100 mg D and 1000 mg Q for 3 days decreased blood SASP components and reduced the number of senescent cells in patients with diabetic kidney disease.^259^ Another clinical study (https://clinicaltrials.gov/ct2/show/record/NCT04313634) investigated the effect of senolytics on skeletal health. One hundred and twenty elderly women were randomized into three groups. The first group received D (100 mg for 2 days) plus Q (1000 mg daily for 3 consecutive days starting every 28 days with five total dosing periods). The second group received 20 mg/kg of fisetin for 3 consecutive days on an intermittent schedule starting every 28 days (five total dosing periods). The third group did not receive any intervention. The per cent change in the serum C‐terminal telopeptide of type I collagen (CTX) (bone resorption marker) and the amino‐terminal propeptide of type I collagen (P1NP) (bone formation marker) for a 20‐week period will be examined in this ongoing study. Results are not available.

## 7 CONCLUSIONS AND FUTURE PERSPECTIVES

Cellular senescence plays an important role in bone homeostasis, with variations in cellular senescence based on the different types of OP. These variations are associated with pathogenic factors, bone turnover rate and systemic metabolism. Understanding the molecular relationship between bone cells and senescence provides for the possible targeting of senescence as a means by which to treat OP. However, there is no general consensus regarding the clinical efficacy of cellular senescence‐associated pharmacological therapy for OP. There are several promising approaches, such as the elimination of senescent cells using senolytic agents or immunotherapy, the removal of specific SASP factors with senomorphics, rejuvenation of stem cells using gene therapy and Chinese herbal treatment. However, there are challenges to clinical intervention in humans. For example, the specific senescent cells and components of the SASP that result in the initiation and progression of OP have not been fully identified. Most senescence modulators are not specific to an individual target, and may affect not only senescent cells but also other cell populations. Much work is required to confirm the crosstalk between cellular senescence and OP in humans before preventive and therapeutic strategies can be applied in the clinic. Moreover, personalized therapies will be required because of differences in pathology, types of OP and bone turnover rates.

## AUTHOR CONTRIBUTIONS

Tiantian Wang and Shishu Huang conceptualized and wrote the outline of the manuscript. All authors reviewed and edited the manuscript. All authors have read and approved the final manuscript.

## CONFLICT OF INTEREST

The authors declare no conflicts of interest.

## Data Availability

Data sharing is not applicable to this article as no new data were created or analyzed in this study." cd_value_code="text
